# Helicopter EMS Transport Outcomes Literature: Annotated Review of Articles Published 2007–2011

**DOI:** 10.1155/2012/876703

**Published:** 2012-01-11

**Authors:** Brandon S. Brown, Korby A. Pogue, Emily Williams, Jesse Hatfield, Matthew Thomas, Annette Arthur, Stephen H. Thomas

**Affiliations:** Department of Emergency Medicine, OU Schusterman Center, University of Oklahoma School of Community Medicine, 4502 East 41st Street, Suite 2E14, Tulsa, OK 74135-2553, USA

## Abstract

Helicopter EMS (HEMS) and its possible association with outcomes improvement continues to be a subject of discussion. As is the case with other scientific discourse, debate over HEMS usefulness should be framed around an evidence-based assessment of the relevant literature. In an effort to facilitate the academic pursuit of assessment of HEMS utility, in late 2000 the National Association of EMS Physicians' (NAEMSP) Air Medical Task Force prepared annotated bibliographies of the HEMS-related outcomes literature. As a result of that work, two review articles, one covering HEMS use in nontrauma and the other in trauma, published in 2002 in *Prehospital Emergency Care* surveyed HEMS outcomes-related literature published between 1980 and mid-2000. The project was extended with two subsequent reviews covering the literature through 2006. This review continues the series, outlining outcomes-associated HEMS literature for the three-year period 2007 through the first half of 2011.

## 1. Introduction

Despite the frequency of HEMS transport, there remains controversy surrounding its use and benefits. In 2002, two annotated bibliographies prepared by the National Association of EMS Physicians' (NAEMSP) Air Medical Task Force addressed the HEMS outcomes-related literature for trauma and nontrauma diagnoses [1, 2]. Although commentary was provided for each article, the bibliographies and their summaries of over 50 studies were intended to serve primarily as a central reference listing to aid parties interested in HEMS research. The bibliography has been updated twice, to cover studies published through 2006 [3, 4]. The current paper aims to extend the previous reviews, assessing outcomes studies published 2007 through the time of this review's preparation, in mid-2011. As with earlier reviews in the series, the article summaries include commentary intended to place the research into perspective. The primary goal of this article, like the prior reviews, is to present the most important HEMS outcomes literature published in the 2007–2011 time frame as an aid to those who wish to explore the evidence basis for HEMS use.

## 2. Methods

A computerized literature search was performed. The search database was the National Library of Medicine's MEDLINE (online Index Medicus), extending from 2007 through 2011. The search methods and terminology used for this review were the same as those employed, and reported, in the previous reviews [1–4]. For the current review, there were over 1500 studies assessed for possible inclusion (by review of title, abstract, or full-length paper).

As noted for the previous reviews, eligibility for article inclusion was usually easy to determine, but there was inevitably some degree of subjectivity. The authors acknowledge that the process of article selection may have excluded some worthy research and emphasize that the attempt to capture all relevant papers probably missed some studies.

The papers that are included in this review are categorized into diagnostic areas. The first category is *Airway*, followed by *Cardiac *and *Costs and Benefits*. The next two categories are *Drowning *and *Pediatrics*. The injury categories follow: *Drowning, Trauma-Scene *(studies limited to scene, or primary, HEMS transport), *Trauma-Scene and Interfacility *(casemix consisting of both primary and secondary missions), and *Trauma-Interfacility *(secondary missions only). For interpretation of the trauma studies, some knowledge of TRISS methodology (survival probability based upon trauma and injury severity scores as well as age and injury mechanism) is helpful. TRISS is outlined in detailed elsewhere [5]. The next category, *Trauma-Scene and Interfacility*, addresses the use of HEMS for a patient population comprising both primary (i.e., scene) and secondary (i.e., interfacility) HEMS missions. The review concludes with *Drowning *and *Cardiac *sections. Within categories, articles are listed chronologically with earlier papers first.

## 3. Airway

### 3.1. Nakstad AR, Heimdal HJ, Strand T, Sandberg M

“Incidence of desaturation during prehospital rapid sequence intubation in a physician-based helicopter emergency service”, *Am* *J* *Emerg* *Med*, 2010 April 30 (epub ahead of print).

**Objective**

This study attempts to establish the incidence of hypoxemia in patients intubated by a physician in a helicopter emergency service.

**Methods**

**Study Design**

This was a prospective, observational study of all RSIs performed by helicopter emergency service physicians during a 12-month period. Hypoxemia was defined as a decrease in S_p_0_2_ to below 90% or a decrease of more than 10% if the initial S_p_0_2_ was less than 90%.

**Setting**

This Norwegian (Oslo) HEMS operates two helicopters 24/7/365 and serves a population of about 2.1 million covering an area of approximately 200 km. Each crew has a pilot, paramedic, and an attending anesthesiologist on board.

**Time Frame**

Data were collected for transports April 1, 2008 to March 31, 2009, including all cases in which drug-assisted Rapid Sequence Intubation (RSI) was provided.

**Patients**

2621 patients were transported; of those, 122 meet criteria for prehospital RSI attempt.

**Results**

Trauma patients comprised the majority of the study cases requiring RSI (79 of 122 patients). There were complete S_p_0_2_ data for 101 (82.8%) of the 122 intubations. On average, RSI took 40.8 seconds with very little difference in times between trauma and medical patients. Neither Cormack-Lehane laryngoscopic view nor operator-defined intubation difficulty was associated with medical versus trauma patient group, and there were also no factors associated with desaturation (which occurred in 11 of 101 patients with complete records).

**Authors' Conclusions**

This study reports a hypoxemia rate of 11.1% in trauma patients and 10.5% in medical patients; these rates are better than many prehospital intubation studies. However, there were 21 cases with incomplete S_p_0_2_ recordings and occult desaturation cannot be ruled out in these cases. The study's relatively low desaturation rates may be explained by training: the study program's HEMS physicians are anesthesiologists with extensive prehospital experience. In this study, inadequate preoxygenation (as defined by preprocedure low saturation) was the only factor associated with desaturation events. Anatomical and technical difficulties increased the time required for intubation but were not associated with increased risk of hypoxemia.

**Commentary**

This study provides compelling evidence that prehospital RSI by a physician-staffed HEMS team yields excellent results which compare favorably to most from the existing (ground EMS) literature.

### 3.2. Le Cong M

“Flying doctor emergency airway registry: a 3-year, prospective, observational study of endotracheal intubation by the Queensland Section of the Royal Flying Doctor Service of Australia”, *Emerg Med J.,* 2010 Sep 15 (epub ahead of print).

**Objective**

This study attempts to describe the profile and success rates of emergency endotracheal intubation conducted by the Queensland Royal Flying Doctor Service (RFDS) aeromedical retrieval team comprising a doctor and a flight nurse.

**Methods**

**Study Design**

Each intubator completed a study questionnaire at the time of each intubation for indications, complications, overall success, drugs utilized, and deployment of rescue airway devices.

**Setting**

The Queensland section of the Australian RFDS has seven aeromedical operations bases and covers about 780,000 sq miles.

**Time Frame**

The questionnaire was distributed from January 1, 2007 to January 1, 2010.

**Results**

76 patients had intubation attempts. 72 were successful attempts. Three failed attempts were managed using Larygneal Mask Airways (LMAs). The 4th failed intubation was managed successfully by simple airway positioning for support. Complications included two cardiac arrests during intubation. Both were resuscitated within 1 minute of arrest.

**Authors' Conclusions**

A 95% intubation success rate is comparable to other studies of Australian aeromedical support. The two cardiac arrests account for nearly the same rate of cardiac arrest as documented in a recent study on ICU cardiac arrest frequency. This study also demonstrates the utility of the LMA device in the retrieval and transport setting, in particular for managing a failed intubation. These findings add to the growing body of prehospital literature on the intubating LMA as a rescue airway device in the field.

**Commentary**

This short report produced an intubation rate and complication rates that appear comparable to much of the HEMS literature, although the binomial exact confidence interval for ETI success rate is wide (87–99%). Furthermore, the nearly 3% rate of peri-ETI cardiac arrest (95% CI, 0.3–9.2%) is higher than expected from reviewing other HEMS data. Interpretation of these results would be aided by inclusion of more patient and provider characteristics.

### 3.3. Sollid SJM, Lossius HM, Soreide E

“Prehospital intubation by anaesthesiologists in patients with severe trauma: an audit of a Norwegian helicopter emergency medical service”, *Scandinavian J Trauma Resuscitation and Emer Med,* 2010; 18 : 30.

**Objective**

This study aims to evaluate the trauma airway management quality and patient safety associated with prehospital endotracheal intubation (ETI) by anesthesiologists.

**Methods**

**Study Design**

This study was a retrospective medical records review.

**Setting**

The setting for this study was Rogaland County, Norway. The county has a population of over 400,000 and is serviced by the Stavanger HEMS. The HEMS operates day and night and the crew is a pilot, medical crewmember, and physician. The physician must have at least 2 years of anesthesiology training. Both helicopter and ground rapid response vehicle (RRV) are available, with RRV being deployed only as a back-up to helicopter services. Patients were transported to Stavanger University Hospital.

**Time Frame**

The study included all patients with severe trauma that were transported by Stavanger HEMS between 1994 and 2005.

**Patients**

Study patients consisted of 1255 trauma cases defined as severe by a score of 4 or higher on the National Committee on Aeronautics severity of injury or illness index (NACA).

**Analysis**

This analysis used independent sample *t*-tests to compare means, the Mann-Whitney *U*-test to compare nonparametric mean values, and 2 × 2 tables with the chi-squared test for proportions.

**Results**

No significant difference was found with respect to patient age, sex, NACA score, RTS, or GCS when comparing helicopter to RRV transport. While the mean time to the scene and scene time were significantly shorter with RRV, there was no significant difference in transport time between helicopter and RRV. There was also no significant difference in resident or specialist treating physician between helicopter and RRV transport. Of the 1255 study cases, 240 (19%) intubation attempts were made prior to hospital arrival and 47 (16% of total intubations) intubations were conducted upon ED arrival. Of the prehospital intubations, a 99.2% success rate was found; 40 of these patients died before hospital arrival. Of the ED intubations, a 100% success rate was found. The median GCS and RTS were both significantly lower for patients who received prehospital intubation (GCS 3, RTS 3.8) compared to those who received ED intubation (GCS 6, RTS 5.0). Of the prehospital intubations, 71 were conducted without any pharmacologic facilitation and 3 procedural complications were reported. By 2005, capnography use had also increased in these patients to 79% (in successful intubations). Prehospital intubations were found to be made more commonly during helicopter missions versus RRV missions. A significantly greater number of patients (78 versus 55%) who received ED intubation were found to be alive at the time of discharge, compared to those who received prehospital intubation. However, no significant differences were found in days in the hospital, in ICU, or on the ventilator.

**Authors' Conclusions**

This study found that anesthesiologist-managed HEMS ETI had a high success rate with few complications. These findings help to confirm the patient safety in prehospital ETI of this setting. However, 43 patients with GCS ranging from 3 to 8 were not intubated in the prehospital setting, instead receiving ED ETI, with no clear explanation for lack of pre-ED ETI. The authors believe that future prospective studies with data collected in a uniform manner could help to provide better evidence for the quality of prehospital airway management.

**Commentary**

This retrospective study showed that there appears to be no patient safety “cost” (and potentially even some benefit) associated with anesthesiologist-managed airways in the prehospital setting. However, there was a significant shortcoming in terms of available information describing the lack of ETI in patients who seemingly should have been intubated in the field but who were not. As the author states, previous studies have shown failure to adhere to prehospital guidelines and this gap in care is an area that warrants further study in the future.

### 3.4. Sollid SJM, Lossius HM, Nakstad AR, Aven T, Soreide E

“Risk assessment of prehospital trauma airway management by anaesthesiologists using the predictive Bayesian approach”, *Scandanavian J Trauma Resuscitation and Emer Med,* 2010; 18 : 22.

**Objective**

This study's objective was to evaluate quality of care in anesthesiologist-headed prehospital airway management and to identify areas in which improvement may be possible.

**Methods**

**Study Design**

This study used information obtained from a retrospective medical records review, along with author expertise and literature review, to conduct a risk assessment along the principle of the Bayesian approach.

**Setting**

The records reviewed for this study were obtained from the Stavanger HEMS of Rogaland County, Norway. This is considered by the authors to be a typical Norwegian HEMS.

**Time Frame**

Risk assessment was conducted with consideration of the above-mentioned patients with severe trauma that were transported by Stavanger HEMS between 1994 and 2005.

**Patients**

Risk assessment was conducted with consideration of the above-mentioned 1255 trauma cases defined as severe by a score of 4 or higher on the National Committee on Aeronautics severity of injury or illness index (NACA).

**Analysis**

This risk assessment was conducted using the Bayesian approach. This study focused on patients who arrived to the ED without intubation despite prehospital indications (referred to as the “top event”). Probability of this event was predicted. Assessment was also used to evaluate the factors leading to the event and consequences following the event. A fault tree was constructed to identify potential causation of this event. Major categories found in the fault tree included indication for airway management not recognized and airway intentionally not secured (both leading to airway management not being attempted) and tracheal tube in esophagus and attempt aborted (both leading to attempted airway management being unsuccessful). Further, risk influencing factors (RIFs) were applied to each of these basic events in the fault tree. These RIFs included culture and attitudes, provider experience and knowledge, and system/protocol compliance. Each of these was given a score of good, average, or poor related to the quality of functioning within the system. An event tree was also constructed to evaluate potential outcomes following the failure to intubate. These potential outcomes were illustrated based on the authors' knowledge of the system and applied to the risk matrix resulting in placement into five categories of probability. Uncertainty factors that could potentially affect risk factors were also identified and categorized into minor, moderate, or major.

**Results**

In this study, four basic events were discussed as contributing to the non-intubation of the patient. As previously mentioned, these are indication for airway management not recognized, airway intentionally not secured, tracheal intubation in the esophagus, or attempt aborted because patient could not be intubated. Additional risk influencing factors including culture and attitudes, system, provider experience and knowledge, and protocol compliance were identified. Based on study information and knowledge of the system, the authors placed a score on each of the RIFs as follows: culture and attitudes: average; system: poor; provider experience and knowledge: average; and protocol compliance: poor. The scored RIFs were then applied to each of the four basic events with each RIF being given an appropriate adjusted weight (scored as a fraction of 1.0) according to how likely the RIF was to influence the basic event. This information was used by authors to calculate a probability of the top event equaling 29% (meaning 29 of 100 cases). Using the event tree to evaluate potential patient outcomes, authors found that the probability of no harm and of possible sequelae with prolonged hospital stay were approximately equal. As applied to the risk matrix, the patient outcome of no harm was found to have a frequency prediction of 1–10 incidences per year. The patient outcome of possible sequelae with prolonged hospital stay was found to have at least 10–50% probability during one year, but likely to have less than 1–10 incidences per year. Patient death was found to have a probability of less than 1% during one year. The uncertainty factors identified included amount of training to maintain airway skills, need for special training in prehospital airway management, impact of patient's condition on consequences, reliability of data recorded in patient charts, and criteria used to decide whether or not patient should be intubated. All of these factors were decided to have at least moderate effect on risk for the top event. However, none of the factors were deemed to have significant sensitivity in predicting the top event.

**Authors' Conclusion**

The authors' risk assessment resulted in a potentially high probability, as high as 29%, of lack of prehospital ETI in patients with indications for prehospital ETI. Evaluation of patient outcomes also revealed high probability for possible sequelae. It was also found that of potential RIFs, changes in system and in culture and attitudes show the greatest potential for improving patient outcomes.

**Commentary**

The risk assessment conducted in this study is certainly not the “usual” HEMS outcomes analysis. The authors have identified an interesting perspective on HEMS and prehospital patient care, in applying rigorous Bayesian analysis to the endpoint that “should've been intubated but was not.” The results themselves are interesting, but the idea of using these methods to assess HEMS care may be the most important message of this paper.

## 4. Cardiac

### 4.1. Blankenship JC, Haldis TA, Wood GC, et al.

“Rapid triage and transport of patients with ST-elevation myocardial infarction for percutaneous coronary intervention (PCI) in a rural health system”, *Am J Cardiol, *2007; 100 : 944–948.

**Objective**

This study examined the endpoints of time savings and health outcomes, to assess the effects of a new triage and HEMS transfer system designed to expedite community hospital evaluation and referral of STEMI patients to a PCI center.

**Methods**

**Study Design**

This study was “ambispective” in that some of the data were assessed retrospectively, and other patients' data were assessed prospectively. In mid-July 2004, retrospective chart review was performed to assess study variables back to January 2004. From mid-2004 through the end of the study period (December 2005), data were entered prospectively. The overall study design was a “before-and-after” approach, in which endpoints of interest were compared prior to, and after, January 2005 institution of new protocols for community hospital STEMI care and expedited HEMS dispatch. In brief, the protocol changes which were effective mid-way through the study included (1) community hospital STEMI care changes emphasizing time savings (e.g., elimination of heparin and nitroglycerin infusions), (2) simultaneous PCI lab and HEMS activation from a single call to the receiving center, and (3) bypass of the receiving center's ED after HEMS transport.

**Setting**

This study took place in central Pennsylvania, with the receiving center (Geisinger Medical Center) serving 37 counties with a population of 2.4 million. Geisinger is a 437-bed rural PCI center and operates 4 helicopters based at 4 sites in central Pennsylvania. HEMS staffing is primarily by a nurse and flight paramedic, with occasional physician crewmembers. The study HEMS service had a high completion rate (97%) for the cardiac patients transported during the study period.

**Time Frame**

This study took place from January 1, 2004 to December 31, 2005.

**Patients**

Patients eligible for inclusion were drawn from STEMI transports to the study center over the study period. Exclusion criteria included transport >12 hours after symptom onset, failure of thrombolytic therapy, or PCI contraindications. Most of the 226 patients comprising the study sample were male (80%), and the mean age was 58.

**Analysis**

The main time endpoint was the proportion of patients with a time interval from initial community hospital presentation to wire-crossing (in the PCI lab) of under 90 minutes. The time periods were reported as medians, and intervals for the two study periods were compared using nonparametric Kruskal-Wallis testing. Other continuous variables were assessed using Students *t*-test. Categorical data were compared using chi-square or Fisher's exact test as appropriate.

**Results**

For the main endpoint (community hospital presentation to wire-crossing time), the “after” period was associated with significantly shorter times (105 versus 205 minutes, *P* = 0.0001). Part of the time savings were achieved by faster HEMS dispatch (from 35 to 16 minutes, *P* = 0.0004), and further time savings were accrued by streamlining time intervals between HEMS dispatch and PCI center arrival (from 56 to 45 minutes, *P* = 0.002). Additional time savings occurred after arrival to the PCI center; receiving center arrival to wire-crossing times decreased from 91 to 29 minutes (*P* = 0.0001). Of the decrease in total time, 37% (36 minutes) was due to some combination of improved efficiency in STEMI diagnosis, HEMS dispatch, and pretransport stabilization. The remaining 63% of time savings were accrued by a combination of bypassing the receiving center ED, simultaneous cath lab activation (at time of HEMS dispatch), and optimization of PCI lab procedures. The proportion of patients with door to wire-crossing times under 90 minutes increased from 0% to 24% (*P* = 0.0001), and the percentage with door to wire-crossing times under 120 minutes also increased (from 2% to 67%, *P* = 0.0001). There were no significant differences between the before and after periods, with regard to the following patient outcomes: death (*P* = 0.28), urgent revascularization (*P* = 0.62), or hospital length of stay (*P* = 0.46).

**Authors' Conclusions**

The authors concluded that in the rural setting the goal of treating STEMI patients within 90 minutes can be achieved for some transfer patients given a rapid diagnosis, triage, and transfer system.

**Commentary**

This study has particular strengths in its clear demonstration of time savings at a number of steps in the diagnosis/transfer process, with potential for clinical significance. While the lack of identification of any health outcomes benefits is appropriately highlighted in the authors' discussion, it is hard to dispute the criticality of the STEMI care surrogate endpoints relating to time. Considering the lack of downside to the types of protocol changes that were adopted by the authors (and which have been previously described by other HEMS services) [6] there seems sufficient evidence to support broader application of these streamlining principles. In addition to the lack of mortality benefit, there were other limitations to the study. Perhaps most importantly, although 14 community hospitals received training in STEMI stabilization and transport streamlining, the final data analysis excluded 6 hospitals since they cumulatively accounted for only 22 transports. Thus, the “real-world” generalizability of the study results may be lessened, since it is not uncommon for a significant proportion of any HEMS service's transports to come from relatively infrequent users.

### 4.2. Pitta SR, Myers LA, Bjerke CM, White RD, Ting HH

“Using prehospital electrocardiograms to improve door-to-balloon time for transferred patients with ST-elevation myocardial infarction”, *Circ Cardiovasc Qual Outcomes, *2010; 3 : 93–97.

**Objective**

The purpose of this study was to demonstrate that in patients transferred from a rural ST-elevation myocardial infarction (STEMI) referral hospital located 50 miles away from a receiving STEMI center, it is possible to achieve the guideline-recommended goal of first medical contact-to-balloon time of less than 90 minutes.

**Methods**

**Study Design**

This study was a case report of an instance of “positive result” during a 7-month period of prehospital ECG and early cardiac catheterization lab activation. A 12-lead prehospital (PH) ECG program was implemented at a rural hospital that had been achieving door 1-to-first PCI device time of 116 minutes. Implementation of a one-day training program allowed for training EMS personnel to acquire and interpret PH ECG for STEMI. A process was developed for EMS personnel to activate a STEMI protocol that included autolaunching HEMS to intercept the patient and transport to an awaiting cardiac catheterization team. The process would only allow the paramedics to activate the STEMI protocol during a definite STEMI, defined as both EMS paramedics and computer interpretation identifying the presence of acute ST elevation.

**Setting**

This took place at a 77-bed rural community hospital in Minnesota, located 50 miles away from the STEMI receiving center.

**Time Frame**

This case occurred during protocol implementation as monitored between February and August 2009.

**Results**

60 PH ECGs were acquired, resulting in one patient identified with a definite STEMI. The remaining 59 cases were categorized as not STEMI. For the patient identified with a definite STEMI, the first medical contact-to-balloon time was 82 minutes. Symptom onset to balloon time was 117 minutes. These times are within the guideline-recommended performance measures for a patient with STEMI transferred for primary PCI. The door 1 in-to-door 1 out time at the STEMI referral hospital was 11 minutes. The on-scene-to PH ECG acquired time was 15 minutes. At 30-day follow-up, the patient did not have any adverse outcomes. His ventricular ejection fraction improved from 39% following PCI to 52% at 30-day follow-up.

**Author's Conclusions**

The clinical case demonstrates the successful use of PH ECG in order to improve door-to-balloon times for transferred patients with STEMI.

**Commentary**

This case report makes the point that, when incorporated into a system of prehospital and cardiology care, HEMS can be a critical contributor to improved outcome. While the single case report is noteworthy, the article is also potentially important in terms of the 59 cases where HEMS was *not* activated (presumably appropriately). With the growing acceptance of surrogate endpoints such as time savings, HEMS investigators and assessors have more tools to evaluate potential contributions of air medical transport.

## 5. Costs and Benefits

### 5.1. Taylor CB, Stevenson M, Stephen J, et al.

“A systematic review of the costs and benefits of helicopter emergency medical services”, *Injury Int J Care Injured,* 2010; 41 : 10–20.

**Objective**

This study's objective was to conduct a systematic review of available literature that included economic evaluations of HEMS.

**Methods**

**Study Design**

This was a retrospective literature review.

**Setting**

The reviewed studies were conducted across several countries including the United States, Canada, Norway, Germany, Finland, and the UK.

**Time Frame**

The reviewed studies were conducted between 1988 and 2007.

**Patients**

The reviewed studies included a wide variety of patients including trauma, cardiac, stroke, and obstetric patients. Reviewed studies included primary transport patients and patients being transferred between facilities.

**Analysis**

The chosen articles were placed into categories based upon primary patient diagnosis (trauma, nontrauma, and nonspecific) and then further divided based on the type of financial analysis provided within the study (cost-analysis, cost-minimization, cost-effectiveness, and cost-benefit). All financial information obtained from the articles was converted to US dollars and adjusted for inflation between the time of the study and January 2008.

**Results**

Fifteen articles were chosen for this literature review. Seven of these studies focused on the transport of trauma patients. These were subdivided into those focused on primary patient transport, interfacility secondary transport, and studies that included both types of patients. One study that focused on primary patient transport showed a 21-fold variation in the annual cost of HEMS between three services (ranging from $115,777 to $2,436,178). Another one estimated annual HEMS operation at $210,221. None of these studies were able to correlate the increased cost of HEMS to increased patient benefits. Of studies that focused on secondary patient transport, one reported the HEMS per mile cost to be $40 while the fixed wing transport cost was $10 (this study also found no increased patient benefit associated with HEMS transport). Of the studies that focused on both primary and secondary transport, one concluded that annual HEMS cost was $3,023,568 and that was broken down to a cost per patient transport of $15,849. This study also reported the cost per patient of ground transport to be 7 times less than HEMS (at $2,145) without clear benefit to patients. However, two studies of this category found HEMS provided patient benefit of prevention of expected deaths following trauma. These studies found cost-effectiveness ratios of $3,292 and $2,227 per year of life saved. Four studies focused on transport of nontrauma patients. One study in stroke patients found a ratio of $40,954 per each additional good outcome (of minimal to no disability). STEMI patients were found to gain 0.7 life years and decrease lifetime care costs by $7,854 when transported to percutaneous coronary intervention by helicopter. One study showed HEMS transport cost of $3,258 per neonate saved when transporting obstetric patients. Finally, four studies focused on the transport of patients categorized as “nonspecific.” Two of these studies found HEMS transport to be less expensive annually than ground transport for a given geographical area. Two other studies found HEMS to increase patient benefits above “usual care” and to have increased societal benefits.

**Authors' Conclusions**

This review demonstrates that while HEMS can be expensive, air medical transport may in fact be found to be cheaper than ground transport—and may improve patient outcome. While the use of HEMS has been long-reported in the setting of trauma, this review demonstrates that a growing base of evidence may also justify its use in nontrauma patients. Differences in the delivery of HEMS and the systems in which it is delivered render direct comparison of ground and HEMS difficult; further studies of cost-effectiveness will be useful.

**Commentary**

This study shows the breadth of approaches to, and results from, financial assessment of HEMS and patient outcomes. With the *caveat *that the results are only as good as the estimates and assumptions (with respect to both costs and benefits), the overview is a useful step toward rigorous definition of HEMS' place in prehospital care.

### 5.2. Rylski B, Berchtold-Herz M, Olschewski M, Zeh W, Schlensak C, Siepe M, Beyersdorf F

“Reducing the ischemic time of donor hearts will decrease morbidity and costs of cardiac transplantations”, *Interactive Cardiovascular and Thoracic Surgery, *2010; 10; 945–947.

**Objective**

The study's objective was to evaluate the effect of total ischemic time (TIT) during cardiac transplantation on length of stay in the intensive care unit (LOS in ICU) and its economic consequences.

**Methods**

**Study Design**

This was a retrospective single center study.

**Setting**

The study took place at the University Medical Center in Freiburg, Germany.

**Time Frame**

All eligible patients were included between 1998 and 2009.

**Patients**

There were 72 recipients included from a group of 195 cardiac transplant patients. Recipients with prior mechanical support and heart-lung transplantation were not included in this study. In order to make the results representative for the common transplant patient, patients were excluded with TIT less than 100 min and longer than 250 min. Patients who died within 30 days were not included because the cause of death during this period is usually due to acute rejection or surgical complications, which are not associated with TIT.

**Analysis**

Graft TIT was defined as the time interval between aortic cross-clamp application in the donor and aortic cross-clamp removal in the recipient. LOS in ICU with respect to TIT and other clinical parameters was investigated with univariate and multivariate linear regression.

**Results**

The mean age of the 72 recipients (56 men, 16 women) was 50.6 ± 13.2 years (range 15–68 years), and the mean age of the donors was 41.5 ± 12.9 (range 11–51 years). The median TIT was 181.2 (range 107–243) min and mean LOS in ICU was 19.8 ± 19 days. Analysis of the correlation coefficient indicates a statistically significant linear relationship between TIT and LOS in ICU (*r* (72) = 0.327, *P* = 0.005). The linearity implies that each 5 min and 38 s of TIT equates to one more day in the ICU. Univariate analysis of factors associated with LOS in ICU showed that among pre- and intraoperative parameters, only ischemic time significantly impacted LOS. Postoperative parameters revealed that renal failure, defined as a compromise in kidney function requiring hemofiltration, was significantly associated with increasing LOS in ICU. In patients with TIT greater than 180 min, the median LOS in ICU increased 1.7-fold (*P* < 0.01), use of hemofiltration due to renal failure increased 3.2-fold (*P* < 0.05), and nitric oxide (NO) use increased 5.2-fold (*P* < 0.05). Intra-aortic balloon pump (IABP) was required by 3 patients with TIT greater than 180 min. Kaplan-Meier survival analysis revealed that the only significant predictor of survival was postoperative renal failure requiring hemofiltration (hazard ratio 9.1, 95% hazard ratio confidence limits 1.6–51.3, *P* = 0.01).

**Authors' Conclusions**

In this study, increased TIT resulted in greater resource utilization due to postoperative complications. The increased use of hemofiltration, IABP, and NO resulted in longer LOS in ICU and escalated financial expense. The use of the fastest possible transport (Learjet, helicopter) would have economic advantages by decreasing TIT. Also, methods such as beating-heart transport using the organ care system should be considered to reduce morbidity and possibly costs by reducing the actual TIT. This study confirms the findings of other studies suggesting that prolonged TIT during heart transplantation does not significantly influence survival.

**Commentary**

This study is admittedly only tangentially related to HEMS. However, as more pressure is appropriately brought to bear on HEMS to demonstrate cost-effectiveness, there is corresponding impetus to broaden the scope of relevant literature. For those HEMS programs participating in these types of transports, it is quite fair to take this study as a starting point, for generating estimates of benefits associated with HEMS and reduction of total ischemia time.

## 6. Drowning

### 6.1. Barbieri S, Feltracco P, Delantone M, et al.

“Helicopter rescue and prehospital care for drowning children: two summer season case studies”, *Minerva Anestesiol,* 2008; 74 : 703–707.

**Objective**

This study examined the neurological outcomes of nonfatal pediatric immersion injuries, in patients with on-scene apnea, who were treated and transported by HEMS.

**Methods**

**Study Design**

This was a retrospective observational report based upon medical records review.

**Setting**

This study took place in the Veneto Region on the coast of Italy. The area has a coastline of 150 km and a summer population of about 2.5 million. The HEMS unit is staffed by an anesthesiologist and a nurse with prehospital training. Patients were transported to a regional hospital with pediatric intensive care expertise.

**Time Frame**

Data were collected during the May-October time periods for two years (2006 and 2007).

**Patients**

Of the 14 pediatric submersion victims, 9 (64%) were rescued from public pools and 5 (36%) were rescued from lakes or rivers. The victims were up to 16 years old with most of the victims being 4 years old or younger (71%). Ten (71%) were male. While most of the incidents were not witnessed, available data identified submersion times ranging from 5 to 15 minutes. All victims were first rescued by bystanders or a BLS paramedic service, and all were in respiratory arrest at the time of initial rescue.

**Analysis**

Analysis was descriptive. Survival rates and neurological outcomes were assessed at time of discharge and at a 3-month follow-up.

**Results**

At the time of HEMS assessment, 8 of 14 (57%) had deep cyanosis and 10 of 14 (71%) remained in cardiocirculatory arrest despite receiving basic life support efforts by bystanders and BLS-level ground ambulance crews. HEMS crews intubated all patients upon air medical team arrival. HEMS also performed ACLS maneuvers on all patients for a period of pretransport time ranging from 10 to 50 minutes; only 2 failed to respond with a perfusing rhythm within 30 minutes. All patients had a perfusing rhythm by the time of loading onto the helicopter. During flight, 5 patients had persistent hypotension requiring fluids and inotropic support. All victims were mildly hypothermic (mean rectal temperature <35°C; range 32–36°C). IV access was attained on all patients. The on-scene GCS was <8 in all cases. During the transport (average flight time, 14 minutes with a range of 8–20 minutes), the children were warmed with protective blankets. On arrival to the hospital, 10 children had a GCS between 10 and 13, and 2 remained <8. While all patients spent 3–6 days in the intensive care unit, survival with complete/normal neurological recovery occurred in every case. Lack of neurological sequelae was confirmed at three-month follow-up in all cases.

**Authors' Conclusions**

The advanced interventions provided by HEMS crews (e.g., airway and hemodynamic support) were responsible for improved outcome. Promptly dispatching a helicopter with a specialized medical crew is worth the expense as it provides an increased chanced of survival.

**Commentary**

In one sense, the series is certainly impressive. The results are attention-getting: 100% neurologically intact survival in a group of 14 patients with cardiorespiratory arrest from near-drowning. On the other hand, the observational nature of the study combines with the lack of a ground EMS control group to attenuate the strength of conclusions about HEMS' impact. It seems likely that, in an area where the choice is either BLS-level ground response or extended practice-scope HEMS response, pediatric immersion injury patients' best chance at optimal outcome may include air medical care.

## 7. Neurosurgery

### 7.1. Walcott BP, Coumans JV, Mian MK, Nahed BV, Kahle KT

“Interfacility helicopter ambulance transport of neurosurgical patients: Observations, utilization, and outcomes from a quaternary care hospital”, *PloS One,* October, 2011; 6 : e26216.

**Objective**

This study examined a series of neurosurgical interfacility transports, to determine whether clinically significant time savings occurred due to use of helicopter transport.

**Methods**

**Study Design**

This was a retrospective observational report based upon medical records review.

**Setting**

The study took place at the Massachusetts General Hospital, a Harvard-affiliated adult and pediatric level I trauma center in Boston that serves as a regional referral center for a wide variety of neurosurgical patients. The specific transporting HEMS units were not identified in the study, but based upon geographical transport patterns, the vast majority of such cases would have been transported by Boston MedFlight, an RN/EMTP-staffed service.

**Time Frame**

Data were collected for patients transported during the year 2008.

**Patients**

Interfacility-transported patients were eligible if they were transferred from an ED, and who were admitted to (or consulted by) neurosurgery with a primary neurosurgical diagnosis. Excluded patients included those who died during transport, or those who were transported to any area of the hospital other than the ED.

**Analysis**

Analysis was descriptive. Endpoints included patient outcomes, neurosurgical interventions and timing of those interventions, and time savings accrued by air transport (as calculated from air transport times compared to GoogleMaps-calculated driving times).

**Results**

Of 167 patients studied, a breadth of neurosurgical disease and injury was seen (ranging from brain tumor to cerebrovascular disease to trauma and other diagnoses). 4 (2%) died, 4 were discharged from the ED, and the remainder were admitted. 34% of the patients had estimated ground transport times under 45 minutes; only 16% of patients had estimated ground transport times exceeding 80 minutes. Median time to non-neurosurgical interventions (cricothyrotomy, thoracotomy, or laparotomy) on 14 patients (8%) was 29 minutes. Fiberoptic bolts for traumatic brain injury were placed in 8 patients, a median 1.0 hours after ED arrival. Including those bolt placements, the overall median time-to-neurosurgery was 3.2 hours; times varied widely depending on the procedure (e.g., for the 2 spine fusion cases, a median 118 hours after ED arrival). Discharge dispositions were home (34%), inpatient rehab (38%), and death/hospice (28%); 6 patients (4%) were discharged home within 24 hours of admission. Overall, 56 patients (34%) in the HEMS transport cohort neither died nor required any invasive procedure at the receiving institution.

**Authors' Conclusions**

Many patients undergoing interfacility HEMS transport are inappropriately triaged to helicopter transport, as evidenced by actual times to intervention at the accepting institution and estimated ground transportation times from the referring institution.

**Commentary**

This is a fascinating study by a group of neurosurgeons from a world-class institution (disclosure: one of this review's authors, ST, has worked closely in the clinical setting, with at least one of the study's authors). The clinical depth these authors bring to the HEMS discussion is noted and appreciated. The results—which to these reviewers clearly demonstrate overall appropriateness and utility of HEMS—are obviously subject to interpretation. The authors' preexisting notions are delineated in their Introduction statement that “We hypothesize that many of these patients are inappropriately triaged to helicopter transport.” Questionable study presumptions include the positions that (1) HEMS benefit is predicated solely on time savings (the authors' own discussion outlines the importance of physiologic critical care management), (2) a posteriori-estimated ground transport times in a metropolitan area are reliable, and (3) HEMS use should be deemed “inappropriate” for any patients who did not undergo emergency neurosurgical intervention (this ignores the well-accepted principle that avoidance of undertriage results in some level of overtriage). The authors do note some of these limitations, as well as pointing out the major problems associated with lack of ground or HEMS record review (the authors never followed up a late-2010 email exchange with Boston MedFlight, to call and discuss the study and to arrange forwarding of records). The authors also correctly identify the most important issue with the paper: absence of a ground-transported comparison cohort. With the understanding that there is always room for ongoing education and improvement (e.g., through telemedicine, as suggested by the authors), this study's results strongly support the appropriateness of HEMS triage and utilization for this population.

## 8. Pediatrics

### 8.1. Gerritse BM, Schalkwijk A, Pelzer BJ, Scheffer GJ, Draaisma JM

“Advanced medical life support procedures in vitally compromised children by a helicopter emergency medical service”, *BMC Emerg Med,* 2010 Mar 8; 10 : 6.

**Objective**

This study's goals were to evaluate the advanced medical interventions performed by EMS and physician-staffed HEMS in vitally compromised children and to examine how often HEMS provided additional medical care which was not or could not be provided by the ground EMS.

**Methods**

**Study Design**

This study employed a prospective cohort analysis for HEMS calls in a region of eastern Netherlands and enrolled all patients aged 16 or younger. The data points recorded for each patient included age, sex, type of incident, physiological parameters, GCS, prehospital treatment given, diagnosis in the emergency department, and survival until 24 hours after hospital admission. Additionally, all patients transported by HEMS received a NACA (National Advisory Committee for Aeronautics) score.

**Setting**

The HEMS Trauma Region Netherlands–East covers one of the 4 HEMS regions in The Netherlands and covers an area of about 10,000 square kilometers in which 4.5 million people reside.

**Time Frame**

Study patients were those transported from 2001 to 2009.

**Patients**

The study included 803 HEMS calls. 245 of these were cancelled before HEMS could arrive. HEMS attended to 558 children, all of whom are included in this study.

**Results**

The 558 patients received 1649 advanced medical procedures and 818 of those procedures required a HEMS physician. 65% of HEMS-transported patients received advanced medical procedures they could not have gotten if transported by ground EMS. Furthermore, HEMS often added value even when performing procedures within the practice scope of ground EMS. For example, intubation by ground EMS paramedics (arriving at the scene before HEMS) was characterized by a 77% success rate in 86 children. In 20 of these patients (23%) emergency correction of the endotracheal tube or ventilator settings was performed by HEMS upon arrival. HEMS intubated 214 children with 100% success rate, and the difference in success rates between EMS and HEMS intubations was statistically significant. Pain management data showed that 77% of patients in need of pain management (as defined by pain medication administration by HEMS) failed to receive analgesia from ground EMS. The youngest patient receiving with pain management by ground EMS was 4 years old; HEMS providers administered analgesia to patients as young as 2 months of age. No detrimental effects of pain management were recorded in this study. Also, the majority of all patients transported by HEMS had a NACA score IV–VII (indicating relatively high acuity).

**Authors' Conclusions**

The HEMS group studied provides essential medical expertise not provided by ground EMS. The authors call for a lower threshold for HEMS activation in any serious incident involving children, preferably based on the type of primary emergency call. Special attention should be paid to the training involved with treating pediatric patients with high NACA scores. Also, the high rate of failed intubations by EMS and the inappropriately infrequent use of analgesia and intraosseous access devices need improvement.

**Commentary**

This study addresses the issue of physician-manned HEMS versus (nonphysician) ground EMS and finds many clear advantages to HEMS. Intubation success rates (100% as compared with 77%) are probably the most critical, but the importance of prehospital pain management as an independent endpoint should not be disregarded. The data are make a convincing case that, in the region studied, physician-staffed HEMS brings substantial benefits to pediatric patients. While no cost-benefit results were reported, it should be remembered that life-saving interventions (e.g., intubation) are financially attractive in children who will lead long healthy lives as a result of HEMS transport.

## 9. Trauma-Scene Transports

### 9.1. McCowan CL, Swanson ER, Thomas F, Handrahan DL

“Outcomes of blunt trauma victims transported by HEMS from rural and urban scenes”, *Prehosp Emerg Care, *2007; 11 : 383–388.

**Objective**

The study's goal was to determine whether HEMS-transported rural scene trauma patients have the same mortality as HEMS-transported urban scene trauma patients.

**Methods**

**Study Design**

This was a retrospective consecutive-case review of records from two HEMS services and three receiving Level I trauma centers. The authors' endpoint analysis incorporated multivariate logistic regression controlling for age, gender, and ISS.

**Setting**

The trauma centers in the study are located in Salt Lake City, Utah; the regional population is 1.4 million. The area's two HEMS services are staffed by paramedic/nurse and nurse/nurse teams (variable depending on patient age).

**Time Frame**

Study patients were those transported during 2001.

**Patients**

The study included 271 urban and 141 rural blunt trauma scene transports. Study patients were aged at least 15 years, and all blunt trauma scene transports were included except for those related to winter resort activities. The authors defined “rural” counties *a priori*, as those with fewer than 99 residents per square mile.

**Results**

There were no significant differences between rural and urban patients' age, gender, or receiving hospital, but the urban group had significantly more autopedestrian/bike victims and the rural group had more “other motorized” vehicle crash victims (e.g., ATV, snowmobile). Urban transports were characterized by shorter scene times (16 versus 21 minutes), shorter flight times (30 versus 79 minutes), and greater likelihood of pre-HEMS IV access and ETI. Urban and rural patients had similar vital signs upon HEMS arrival, but the former group had lower GCS and TS. The main endpoint analyses found that rural and urban patients outcomes were similar with respect to hospital and ICU length of stay, ED death, or inpatient mortality; there were also no differences in discharge status dispositions.

**Authors' Conclusions**

After controlling for age, gender, and ISS, there were no significant mortality differences between rural and urban scene trauma patients undergoing HEMS transport. The lack of mortality difference was also present when analysis was limited to motor vehicle and motorcycle crashes.

**Commentary**

As the stack of TRISS-based studies demonstrating HEMS' trauma outcomes improvement grows, there is shrinking incremental benefit of adding another such study onto the pile. Thus, novel approaches to assessing for HEMS benefit are particularly valuable. These authors took a unique and clever approach to the outcomes assessment problem, taking as their foundation the well-known fact that trauma in the rural setting is associated with worse outcome. There is always potential for residual confounding (e.g., by acuity), but the Utah group went to great lengths to minimize study flaws. The authors' discussion incorporates many points of interest and relevance, but the bottom line is that HEMS use appears to be an “equalizer” for rural trauma patients—air transport eliminated the rural/urban trauma outcomes differences for both mortality and nonmortality endpoints.

### 9.2. Davis DP, Peay J, Good B, et al.

“Air medical response to traumatic brain injury: A computer learning algorithm analysis”, *J Trauma,* 2008; 64 : 889–897.

**Objective**

The study's goal was to determine whether air medical transport of head-injured patients from trauma scenes was associated with mortality benefit.

**Methods**

**Study Design**

This retrospective study generated predictive models using artificial neural network (ANN), support vector machine (SVM), and decision tree methods. ANN was used to calculate differential survival (actual versus predicted) for each patient, and SVM used chi-squared testing to compare (between air- and ground-transported patients) the ratios of unexpected survivors to unexpected deaths. Decision tree analysis was used to explore the indications for air transport.

**Setting**

This was a registry-based analysis from the San Diego County Trauma Registry.

**Time Frame**

Study patients were those transported during 1990–2003.

**Patients**

The study included 11,961 patients with head AIS at least 3; 3,023 were transported by air and the others by ground ambulance (usually with paramedic-level care).

**Results**

All three algorithms generated by the study's methodology predicted a survival benefit associated with air transport across all patients. The benefit was most pronounced in cases with higher acuity as denoted by GCS, ISS, head AIS, or hypotension.

**Authors' Conclusions**

Air medical response confers a survival advantage in traumatic brain injury (TBI).

**Commentary**

This was not a “TRISS” study, but some parts of the methodology are reminiscent of that approach. Specifically, the ability of the study to identify unexpected survivors (and unexpected deaths) is an important function. The authors' study, while necessarily complex, appears to represent an unbiased, reproducible, valid (as tested statistically) mechanism for identifying the differential effect of transport mode on survival outcome. Based upon means of the three best ANN models, the differential survival attributable to HEMS as compared to ground transport was calculated to be 3.6 per 100 (95% CI 3.4 to 3.9) for all patients studied. When the study group was AIS at least 4, the survival benefit rose to 5.7/100; in patients with GCS between 3 and 8 the benefit was 7.1/100 patients. Since these same authors have also demonstrated that HEMS improves nonmortality outcome (i.e., functional survival), the results of the current study's methods are a useful complement to the existing body of evidence that strongly suggests HEMS impacts TBI outcome.

### 9.3. Ringburg AN, Spanjersberg WR, Frankema SPG, et al.

“Helicopter Emergency Medical Services (HEMS): Impact on on-scene times”, *J Trauma, *2007; 63 : 258–262.

**Objective**

The study's objective was to compare prehospital on-scene times (OSTs) for patients treated by nurse-staffed ground emergency medical services (EMS) with OSTs for patients treated by a combination of ground EMS and physician-staffed helicopter emergency medical services (HEMSs). Due to relatively short ground transport times from scenes, the responding HEMS unit rarely performs actual patient transports; HEMS crews perform patient stabilization and attend the patients while *en route *to hospital. A second aim was to investigate the relationship between length of OST and mortality.

**Methods**

**Study Design**

The study was a trauma registry study using regression analysis to compare EMS to EMS/HEMS-treated patients.

**Setting**

The study patients were those at trauma scenes in the area served by HEMS based out of Rotterdam, in The Netherlands. Patients were transferred to a high-level trauma center (Erasmus University Hospital).

**Time Frame**

Study patients were cared for between January 2002 and 2004.

**Patients**

Study patients were all (*n* = 1, 457) adult (>15 years old) trauma scene transports to Erasmus during the study period: 260 (18%) in the HEMS group and 1,197 (82%) in the EMS group.

**Analysis**

Mean prehospital on-scene times between groups were compared using Student's *t-*tests. A custom-fitted regression model was defined to compensate for potential selection bias. All commonly used predictive variables were evaluated for their contribution to the model. The variables-Revised Trauma Score (RTS), age, Injury Severity Score (ISS), whether the trauma occurred inside or outside the uniform daylight period, and mechanism of injury were found to have significant predictive value and were fitted into the model. Logistic regression models were used to analyze the influence of OST on mortality.

**Results**

The number of trauma patients included for analysis was 1,457. HEMS patients had longer mean OSTs (35.4 versus 24.6 minutes; *P* < 0.001). After correction for patient and trauma characteristics (including RTS, age, ISS, daytime/nighttime, mechanism of injury), the difference in OSTs between the groups was 9 minutes (*P* < 0.001). Unadjusted logistic regression suggested a 20% higher chance of dying associated with increased OST by 10 minutes (OR, 1.2; *P* = 0.001). However, adjusted analysis found that for HEMS-attended patients the effect of OST on mortality was eliminated (OR 1.0, *P* = 0.89).

**Authors' Conclusions**

Combined EMS/HEMS assistance at an injury scene is associated with longer OST, but this prolongation of OST did not have the anticipated (undesirable) effect on mortality. HEMS response to a trauma scene is associated with earlier provision of critical care interventions that, while increasing prehospital time, provide more rapid “golden hour” procedures that improve mortality and eliminate adverse effect from prolonged OSTs.

**Commentary**

This paper, from well-published and methodologically accomplished investigators in The Netherlands, addresses HEMS outcomes in an indirect manner. The arrangement of HEMS response and subsequent ground transport, unusual in the U.S., has been shown to work well in these authors' country [7]. Regardless of how patients get to the hospital, the important HEMS intervention is—in the judgment of the study authors—getting the experienced crews to the patients. The fact that the provision of advanced care in the prehospital setting negated the adverse outcomes expected due to prolonged on-scene time could be interpreted in two ways. It could be said that the savings of the time associated with more prehospital procedures would get the patients to the trauma center faster, and their “golden hour” interventions could be instituted earlier. This argument may hold true for patients whose ground transport times would be anticipated to be shorter than the 9-minute time prolongation associated with HEMS crew interventions. For other patients, however, the authors' results do seem to make the case that a little prolongation of on-scene time in their setting allows for earlier institution of life-saving interventions.

### 9.4. Cudnik MT, Newgard CD, Wand H, Bangs C, Herrington R

“Distance impacts mortality in trauma patients with an intubation attempt”, *Prehosp Emerg Care,* 2008; 12 : 459–466.

**Objective**

Out-of-hospital endotracheal intubation (ETI) has been associated with adverse outcomes; whether transport distance changes this relationship is unclear. The authors sought to determine whether there was an association between transport distance and prehospital ETI's impact on outcome.

**Methods**

**Study Design**

The study was a retrospective analysis of a consecutive-case adult cohort.

**Setting**

The study covered 19 counties in Oregon's northwestern portion (including the greater Portland metropolitan region).

**Time Frame**

Study patients were accrued to cover 2000–2003.

**Patients**

Study patients were consecutive (*n* = 8, 786) adult (>14 years old) trauma scene transports to the study center over the study years. Of these patients, 534 (6%) underwent prehospital ETI, 307 (57.5%) with rapid-sequence induction (RSI), and 227 (42.5%) without RSI.

**Analysis**

Multivariate logistic regression analysis was used to evaluate the association between prehospital ETI and mortality, and also to assess for effect modification (i.e., statistical significance of an interaction term) between transport distance and ETI. The authors used propensity scoring (for ETI likelihood) and adjusted for potential patient and injury-type confounders, creating estimates for ETI-associated mortality odds ratios (ORs).

**Results**

Of 8,786 patients analyzed, 534 (6%) underwent prehospital ETI. Helicopter transport was used for 962 (10.9% of 8786) patients; 211 of 962 (21.9%) were intubated by HEMS crews. Patients requiring ETI tended to have lower GCS scores, higher injury acuity, and worse outcomes than nonintubated patients. After adjusting for potential confounders and the propensity to be intubated, the authors found prehospital ETI to be associated with an increased mortality (OR 2.1, 95% CI 1.3–3.2) and increased risk of complications (OR 2.1, 95% CI 1.5–2.9). The authors found an association between transport distance and ETI-associated mortality: shorter transport-distance patients had the highest ETI-associated odds of death (OR 4.0, 95% CI 2.1–7.6) and risk of complications (OR 4.1 CI 2.4–7.1). More importantly for this review, the authors found a strong across-the-board (i.e., ETI and non-ETI patients) association between HEMS (versus ground) transport and improved survival (OR 0.3, 95% CI 0.2–0.5).

**Authors' Conclusions**

Prehospital ETI is associated with an increase in mortality among trauma patients at all distances from Level 1 trauma centers, with the greatest prehospital ETI-associated mortality risk increase occurring for patients who are relatively close to the trauma center. Helicopter transport is associated with improved survival in trauma patients, even after adjustment for ETI status and transport distance.

**Commentary**

As noted in previous reviews of the HEMS literature, studies like this one—those that were never intended to assess HEMS' impact on survival—are a double-edged sword. It is vital to avoid overinterpretation of a “secondary” result that was outside the *a priori *intent of the study design. However, the consistently strong association (in these authors' regression models) between survival improvement and HEMS transport should not be ignored. The finding may have been “incidental,” but the HEMS term's statistical and clinical significance in such a methodologically sound study is noteworthy—and perhaps even more given the low likelihood of author bias toward either side of the HEMS debate.

### 9.5. Shepard M, Trethewy C, Kennedy J, Davis L

“Helicopter use in rural trauma”, *Emerg Med Australas,* 2008; 20 : 494–499.

**Objective**

This study's main objectives were descriptive. The investigators also set out to assess whether there were any time savings or outcome advantages accrued by HEMS use or physician staffing, respectively.

**Methods**

**Study Design**

This study was a retrospective medical records review.

**Setting**

The study patients were transported by the Hunter New England Rescue Helicopter Service, which serves the areas northwest of New South Wales in Australia. The HEMS staffing model usually comprises nonphysician prehospital personnel, with the addition of physician crew based upon pretransport judgment that patients might need more advanced care. The physicians who participated in HEMS transports were specially trained doctors with prehospital experience. Patients were transported to Tamworth Rural Referral Hospital in New South Wales.

**Time Frame**

The study included all HEMS trauma missions from January 2004 through November 2006.

**Patients**

Study patients included 129 HEMS scene transports, nearly all for blunt trauma. Of these patients 50 (29%) had an ISS >15 and the average ISS for the study cohort was 12.3.

**Analysis**

The analyses included descriptive approaches as well as univariate assessments. In order to determine whether physician staffing was appropriately triaged, ISS scores were compared between physician-staffed and nonphysician-staffed flights. There were insufficient deaths (2) for meaningful mortality analysis. For the endpoint of transport time, Global Positioning System (GPS) devices were used to estimate road travel times. Times were then compared using Student's *t-*test, within three one-way transport distance categories (<50 km, 50–100 km, >100 km).

**Results**

There was no significant difference between ISS for patients on physician-staffed versus nonphysician-staffed transports. Overall, the average time from dispatch to trauma scene arrival was 48.6 min and the average on-scene time was 50.3 min. The average distance from scene back to the receiving hospital was 160.4 nautical miles. When the times required for HEMS versus (calculated) ground vehicle transport times were compared in the three *a priori-*defined distance groups, the only subcategory with a HEMS time advantage was for distance exceeding 100 km. For transport distances of 50–100 km, there was no time difference between HEMS and ground transport; ground transport was significantly faster when transport distances were less than 50 km.

**Authors' Conclusions**

The conclusions most relevant to this review were as follows: (1) addition of a physician to a HEMS crew has no mortality impact, and (2) HEMS response to a trauma scene within 100 km of the receiving hospital does not result in faster time-to-trauma center.

**Commentary**

The ability to draw definitive conclusions from this dataset is limited by a number of factors. There is potential for selection bias. Furthermore, the very low mortality of the HEMS patients was correctly identified by the authors as problematic. First, there is the obvious fact that such a low death rate indicates room for improvement in triage. Second, the low mortality precludes meaningful assessment of associations between survival and time-distance or staffing variables. The pitfalls of using computers to retrospectively estimate ground transport times have been iterated in previous discussions [3]. Overall, the study should prompt further consideration as to whether HEMS is really necessary for responses within 100 km, in areas that are similar to the study region.

### 9.6. Berlot G, La Fata C, Bacer B, et al.

“Influence of prehospital treatment on the outcome of patients with severe blunt traumatic brain injury: a single-centre study”, *Eur J Emerg Med,* 2009; 16 : 312–317.

**Objective**

This study's objective was to compare the outcomes of TBI patients who were transported by HEMS versus ground ambulance.

**Methods**

**Study Design**

This was a retrospective medical records review.

**Setting**

This study took place in northeastern Italy in a region called Fruili-Venezia Giulia (FVG), with a population of about 1.5 million. Patients with TBI were delivered to one of two regional neurotrauma centers. HEMS operates during daylight hours, with a crew of two nurses and an anesthesiologist with prehospital experience. The comparator ground ambulance service, staffed by two nurses (with occasional addition of a nonspecialized physician), covers a much more limited geographic area around Trieste, the capital of FVG.

**Time Frame**

This study included patients transported from January 2002 to December 2007.

**Patients**

Study patients were 194 cases of scene response to patients who were ultimately found to have ISS at least 15 and a head abbreviated ISS (aISS_head_) of at least 9. The HEMS and ground ambulance groups consisted of 89 and 105 patients, respectively.

**Analysis**

Initial univariate analyses were performed to assess for baseline differences between HEMS and ground patients. Subsequently, the primary study outcomes of mortality and discharge condition were assessed for HEMS versus ground. The discharge conditions were divided *a priori *into 3 groups: (1) alive with no deficit or minor neurological symptoms, (2) alive with severe neurological disabilities (e.g., persistent vegetative status, hemiplegia), and (3) deceased. Secondary outcomes included (among others) prehospital times, hypotension upon arrival at the receiving hospital ED, in-hospital times (including time from receiving hospital arrival to arrival at ICU or operating suite), and ICU and hospital lengths of stay. Statistical analyses included Student's *t*-test and the Wilcoxon rank-sum test for continuous variables, with employment of chi-square analysis and Fisher's exact test for categorical variables.

**Results**

Univariate analysis identified no statistical differences between HEMS and ground groups with respect to on-scene GCS, ISS, aISS_head_, or age. The primary endpoints of mortality and neurological outcome both favored HEMS. Overall, air-transported patients had significantly lower mortality than ground transported patients (21% versus 25%). HEMS patients also had better neurological outcome. Within the group of surviving patients, HEMS patients were significantly less likely than ground patients to have severe neurological deficit (25% versus 31%). Analysis of the secondary endpoints showed that HEMS patients had significantly longer prereceiving center times (66 versus 38 minutes), but that receiving center stabilization time (i.e., time in ED pre-ICU or preoperating room) was significantly shorter for HEMS patients (99 versus 115 minutes). HEMS patients were significantly more likely to undergo prehospital intubation (82% versus 38%) and had twice as many intravenous lines placed on a per-patient basis. In terms of minimizing potential for secondary brain injury, ED arrival blood pressure was significantly higher in the air-transported cohort (mean 133 versus mean 110, *P* < 0.001). There were no differences between air and ground transported patients, with respect to number of neurological procedures, duration of intubation, or ICU or hospital lengths of stay.

**Authors' Conclusions**

The authors believe that the better outcomes, in terms of both survival and neurological condition, that were seen in the HEMS group were due largely to enhanced skills and experience of the air medical teams. Longer prereceiving center times for HEMS patients were offset by the increased number of interventions provided during the prehospital phase. In patients with severe TBI, the concept of the “golden hour” should be modified to adjust for interventions that occur during that critical time period.

**Commentary**

This study, like some others from the same region of Italy [8–10], makes a strong case for HEMS' salutary outcome effect in an “apples-to-oranges” assessment (of greater capability HEMS versus lesser capability ground EMS). The study has limitations—among them the lack of multivariate analysis and the nonadjustment for prehospital times in the reporting of some EMS interventions (i.e., reporting of total volume of fluid resuscitation, rather than volume per hour). In terms of secondary outcomes, the potential for secondary brain injury (which is indeed an important surrogate marker) may have been better evaluated by assessing incidence of hypotension rather than mean blood pressure. Overall, however, the reduction in mortality and the favorable effects of HEMS upon neurological outcome combine effectively with other data suggesting that HEMS improves outcome in TBI patients [11, 12].

### 9.7. Brown JB, Stassen NA, Bankey PE, Sangosanya AT, Cheng JD, Gestring ML

“Helicopters and the civilian trauma system: National utilization patterns demonstrate improved outcomes after traumatic injury”, *J Trauma,* 2010; 69 : 1030–1036.

**Objective**

This study's objective was to compare outcomes between helicopter transport (HT) and ground transport (GT) of injured patients from the scene of injury.

**Methods**

**Study Design**

This was a retrospective study using the National Trauma Databank version 8.

**Setting**

The patient sample was nationwide.

**Time Frame**

Data from 2007 were analyzed.

**Patients**

Patients transported directly to a trauma center from the scene of injury by HT or GT were included. Interfacility transfer patients and patients who were dead on arrival were excluded.

**Analysis**

Stepwise logistic regression was used to determine whether transport modality was a predictor of survival or discharge to home. Stepwise univariate analysis identified all covariates for inclusion in the regression model.

**Results**

In this study, 258,287 patients were transported by helicopter (16%) or ground (84%). Mean ISS was higher in HT patients (15.9 ± 12.3 versus 10.2 ± 9.5, *P* < 0.01), as was the percentage of patients with ISS >15 (42.6% versus 20.8%; OR 2.83; 95% CI 2.76 to 2.89). HT patients had higher rates of intensive care unit admission (43.5% versus 22.9%; OR, 2.58 with 95% CI, 2.53 to 2.64), mechanical ventilation (20.8% versus 7.4%; OR, 3.30 with 95% CI 3.21 to 3.40), and requirement for emergent surgical intervention (18.9% versus 12.7%, OR 1.60; 95% CI 1.56 to 1.65). 14.7% of HT subjects versus 25% of GT subjects were discharged alive within 24 hours of admission to the hospital. However, HT became an independent predictor of survival (OR 1.22, 95% CI 1.17 to 1.27) and discharge to home (OR, 1.05, 95% CI 1.02 to 1.07) when compared with GT after adjustment for patient, injury, and hospital covariates.

**Authors' Conclusions**

HT increased the likelihood of survival and discharge to home after treatment; thus air medical transport has merit and improves outcome. The authors also state that patients being selected for HT “are appropriately sicker and are more likely to use trauma center resources than those transported by ground ambulance.” In addition, the authors note that because the injury severity drops off more drastically for HT than GT as the transport time increases, other factors such as distance and geography rather than injury severity alone are influencing the decision to use HT. The authors conclude that although overtriage continues to be an issue requiring attention by individual trauma systems, it is not as profound a problem as previously reported.

**Commentary**

The largest study in the HEMS literature (HEMS *n* 41,987, GEMS *n* 216,400), this analysis of National Trauma Data Bank (NTDB) data identified a 22% improvement in mortality associated with HEMS as compared to ground transport, for scene trauma patients of all ages/mechanisms (only dead-on-arrival patients were excluded). The study was able to incorporate a broad array of covariates: age, gender, insurance status, mechanism of injury, prehospital times (calculated for HEMS due to straight-line travel and assuming 150 mph transport speed; unavailable for GEMS), Injury Severity Score (ISS), Glasgow Coma Score (GCS), admission systolic blood pressure and respiratory rate, hospital and intensive care unit admission and length-of-stay, mechanical ventilation duration, Emergency Department (ED) and hospital disposition, and hospital trauma center designation. In addition to the outcomes advantage, significant findings included high acuity for HEMS patients nationwide (nearly half requiring ICU, a fifth intubated for an average of a week, a fifth requiring urgent operative intervention)—the authors write that “On a national level, patients being selected for HEMS are appropriately sicker and are more likely to use trauma center resources than those transported by ground ambulance.” The study also found, in terms of triage, that ISS dropped off only as distance from the trauma center increased—so HEMS is being appropriately used to get patients in timely fashion, to trauma centers, for logistics reasons when this is necessary. The study reported a last counterargument to “overutilization” that <15% of HEMS patients nationwide were discharged within 24 hours.

### 9.8. Nakstad AR, Strand T, and Sandberg M

“Landing sites and intubation may influence helicopter Emergency Medical Services on-scene time”, *J Emerg Med, *2010; Aug 23 (epub ahead of print).

**Objective**

The purpose of this study was to evaluate how landing site and rapid sequence intubation (RSI) affect on-scene time (OST).

**Methods**

**Study Design**

This was a prospective observational study.

**Setting**

The Oslo University Hospital HEMS is an anesthesiologist-staffed service that operates in a mixed urban and rural area in southern Norway. The HEMS does not operate with fixed landing sites. A landing site is chosen by HEMS that is as close to the scene of the accident as possible.

**Time Frame**

Data were gathered from January 1 to December 31, 2002.

**Patients**

All trauma mission patients were included in this study. Interhospital transports of trauma patients and primary missions to trauma patients in which the crew responded by the rapid-response car were excluded from this study. Indications for RSI were actual or potential airway compromise, ventilatory failure, or a Glasgow Coma-Scale score of <9 secondary to head injury. Ketamine was used as the induction agent followed by succinylcholine. Tube position was verified clinically and by capnography.

**Results**

During the study period, HEMS executed 252 trauma missions involving 298 patients. Information regarding the landing site was available for 214 (84.9%) of the missions. Landing closer than 50 m from the accident was possible in 75% of recorded cases. In 18% of the missions, the helicopter landed between 50 and 200 meters from the accident. The distance exceeded 200 m in 7% of the cases, and additional ground transport was used in most of those cases. The HEMS anesthesiologist performed RSI in 48 patients before the start of transport. Head injury with Glasgow Coma-Scale score <9 was the indication for RSI in 56.3% of the cases. The median OST was 14.5 min when the patient was not intubated and 22.7 min for HEMS-intubated patients. The difference between the mean on-scene times was 8.2 min (*P* < 0.001). The NACA (National Advisory Committee of Aeronautics) revealed a marked difference in severity between the injured patients who were endotracheally intubated on-site and the spontaneously breathing patients who were transported by HEMS.

**Authors' Conclusions**

The author states that unpublished results from the Oslo University Hospital HEMS show that transfer of a patient between ground ambulance and helicopter takes between 5 and 10 min. Thus, the potential 5–10 min delay is avoided in a large majority of cases (when helicopters land close) since ground transport is unnecessary. This is an argument against the use of fixed, predetermined landing sites. Furthermore, the increased OST of 8.2 min is on the same order of magnitude as found in other studies. The time required for other prehospital procedures such as positioning, suctioning, and establishing intravenous access cannot be separated because an RSI is never performed in isolation. If an RSI had not been performed, it would have been necessary to manage the patients' airways by other means such as supraglottic device or bag-mask-ventilation. Both of these methods not only may shorten OST but also may increase the risk of airway compromise.

**Commentary**

On-scene time is an important “outcome” for prehospital studies (including HEMS investigations) and minimization of on-scene time is a laudable goal. The use of on-site helipads for receiving hospitals has received attention from organizations such as the National Association of EMS Physicians (NAEMSP). On-site hospital helipads are preferred due to both the savings of time (the endpoint of the current study) and also the elimination of an extra physical transfer of the patient. This study makes a strong case that the extra transport leg associated with HEMS landing distant from trauma scenes is just as undesirable as is the aircraft's landing at a remote helipad at the receiving hospital end. Of course, the reason for predetermined landing zones is largely related to safety, and any adjustment of scene landing sites must take this all-important variable into account.

### 9.9. Littlewood N, Parker A, Hearns S, Corfield A

“The UK helicopter ambulance tasking study”, *Injury,* 2010; 41(1) : 27–9.

**Objective**

The aim of this study is to establish and compare the tasking criteria, dispatch arrangements, and crew configuration for all helicopter ambulance services in the United Kingdom.

**Methods**

**Study Design**

The authors employed a structured telephone interview of all the helicopter ambulance services in the United Kingdom. Information was gained from the duty paramedic or doctor and was supported by hard copy documentation for each service's tasking criteria by 14 of the 16 services surveyed. Key types of information requested included number of helicopters, annual number of missions, crew configuration, dispatcher type, existence of dedicated HEMS tasking desk in ambulance control center, and tasking criteria.

**Time Frame**

The interviews were conducted in the spring of 2008.

**Results**

All 16 helicopter ambulance services responded and all information requested from each was complete. The services operate between 1 and 3 helicopters each and have an annual mission frequency between 620 and 2000. Of the 16 services, nine are crewed by paramedics, three have a doctor full-time, and four have a paramedic crew with a doctor on board for a variable proportion of missions. Only six of the 16 have dedicated helicopter ambulance dispatch desks. Only two of the services have medically trained dispatchers (both are paramedics). A total of 67 tasking criteria were identified and divided into six groups: mechanism of injury, anatomical injury type, physiology of injured patients, nontraumatic medical conditions, location, and miscellaneous.

**Authors' Conclusion**

Wide variation in criteria for tasking, crew configuration, and dispatch arrangements exist between the helicopter ambulance services in the UK. These variations may be due to local geographic factors, economic issues, or the availability of a flight crew doctor. The lack of a formal audit or validation criteria may also play a role in the variability observed. The authors cite a lack of evidence-based guidelines for HEMS as another possible reason for the nonuniformity observed in their survey results. They also argued that tasking for paramedic-only crewed aircraft should be focused on remote or inaccessible locations where prolonged ambulance access times would hinder patient care. In order to develop helicopter ambulance service tasking criteria, the cases currently being attended need to be analyzed in terms of shortened response times and improvements in direct triage to definitive care. For physician-crewed aircraft, the incidence of critical care interventions should be analyzed along with the tasking criteria utilized in their activations to determine the most efficient use of those specially trained crews. A nonstandardized approach to air ambulance dispatch criteria and crew configuration is concerning given the financial implications of unwarranted air ambulance use along with the physical risks of aeromedical transport.

**Commentary**

This paper actually focuses on dispatch. It is included in an “outcomes” overview because of its mention of “lack of evidence-based guidelines for HEMS” as a potential explanation for nonstandardized HEMS dispatch. The paper's authors correctly point out that nonstandardization of HEMS use is not defensible. While the available evidence for HEMS suffers from much the same imprecision as do the data addressing many other prehospital (and in-hospital) interventions, it is clearly the case that some level of dispatch standardization, is better than none. The available HEMS outcomes evidence as discussed in this overview (and its preceding editions) is *prima facie* evidence that there are *some *data upon which to base determinations as to how to appropriately use HEMS. The argument that “there's no evidence” can no longer be accepted as a basis for failure to establish guidelines for utilization of the HEMS resource.

### 9.10. Sullivent EE, Faul M, Wald MM

“Reduced mortality in injured adults transported by HEMS”, *Prehosp Emerg Care,* 2011; 15 : 295–302.

**Objective**

This study's objective was to determine whether the mode of transport of scene trauma patients affects mortality.

**Methods**

**Study Design**

This is a retrospective study using the National Sample Program of the 2007 National Trauma Data Bank.

**Setting**

The patient sample was nationwide.

**Time Frame**

Data from 2007 were used.

**Patients**

Patients transported directly to a trauma center from the injury scene were included. Interfacility transfer patients, patients aged <18, and patients transported by means other than helicopter or ground ambulance (fixed-wing, walk-in, private vehicle, public transportation, law enforcement) were excluded. Only records from facilities in which ≥80% of the patients had all three Revised Trauma Score (RTS) physiologic data were included.

**Analysis**

Standard logistic regression model without stepwise procedures was used to assess the association of mortality with mode of EMS transport after controlling for potential cofounders. The variance inflation factor test was used to detect multicollinearity among all of the dependent variables. Subanalyses of those aged 18–54 years and those aged 55 years were performed to assess outcome differences.

**Results**

In this study, 56,744 patients were transported by helicopter (17.7%) or ground (82.3%). The odds of death were 39% lower in those transported by HEMS compared to those transported by ground ambulance (OR = 0.61, 95% CI 0.54 to 0.69). Among those aged 18–54 years the odds of death were 49% lower among helicopter-transferred patients compared to those transferred by ground ambulance (OR = 0.51, 95% CI 0.44 to 0.60). Among those aged ≥55 years, the odds of death were not significantly different (AOR = 0.92, 95% CI 0.74 to 1.13). Among all transports, male patients had a higher odds of death compared to female patients (OR = 1.23, 95% CI 1.10 to 1.38).

**Authors' Conclusions**

The use of HEMS for the transport of trauma patients is associated with reduced mortality in adult patients under age 55 years. The 39% reduction in odds of mortality in adults transferred by HEMS compared to ground ambulance is greater than the 20–30% reduction in mortality reported in most of the previous studies. However, HEMS did not improve mortality in adults aged ≥55 years. Based on this latter finding, the authors suggest that the transport mode may not provide a positive effect on mortality in injured older adults. The establishment of a method for selecting those patients who would most benefit from helicopter transfer is expected to enhance the reduction in mortality shown in this study.

**Commentary**

These authors analyzed the 2007 NTDB data, although the overall *n* was slightly different from the Brown et al.study above due to differing methodology (e.g., different treatment of cases with missing data). Multivariate logistic regression adjusting for age, gender, ISS, and RTS identified a 39% reduction in mortality associated with HEMS. Since the data analyzed were a large subset of the same database used in the study published a few months earlier by Brown et al., this study's findings are confirmatory, if unsurprising. Noteworthy for this study is that when analysis focused on older adults (age >55), the statistical significance of HEMS' mortality benefit was lost (odds ratio 0.92, 95% CI 0.74–1.13). It is quite premature to conclude that HEMS should not be used for older patients, but the finding of limited benefit in those over 55 should spur further investigation of air medical deployment for geriatric trauma.

### 9.11. Stewart KE, Cowan LD, Thompson DM, Sacra JC, Albrecht R

“Association of direct helicopter versus ground transport and in-hospital mortality in trauma patients: a propensity score analysis”, *Acad Emerg Med*, 2011; 18: 1208–1216.

**Objective**

The study's goal was to determine whether, for scene trauma transport, there was an association between air versus ground transport mode and mortality.

**Methods**

**Study Design**

This was a retrospective consecutive-case review of records from a statewide trauma registry (with mandatory data submission for all hospitals). Propensity scoring developed with logistic regression was used to account for variables potentially confounding the association, which was tested with a Cox proportional hazards model. The model included propensity scores as well as ISS, initial RTS, and transport distance. The authors' endpoint analysis focused on 2-week mortality.

**Setting**

This was a statewide study, conducted in the U.S.' southwestern state of Oklahoma. HEMS services operating in the state were RN/EMTP-staffed.

**Patients**

Patients were 10,184 scene patients, transported 2005 through 2008, by either ground (*n* 7467) or air (*n* 2717) EMS, directly to either the state's single Level I trauma center (in Oklahoma City) or to one of the state's two Level II centers (100 miles from Oklahoma City, in Tulsa).

**Results**

The overall hazard for 2-week mortality was 33% lower in HEMS as compared to ground EMS patients, when adjusting for propensity, ISS, RTS, and transport distance. The benefit was greatest in patients with mid-range RTS (39% mortality reduction for RTS between 3 and 7). For the 75% of patients who had normal vital signs at the scene, the hazard ratio point estimate for mortality reduction was 35% but statistical significance was not reached due to this group's overall low mortality (and wider confidence intervals). In the patients with very poor RTS scores (3 or less), there was no difference in mortality between HEMS and ground transport.

**Authors' Conclusions**

After controlling for a number of factors, and using a model which appeared to account for multiple potential confounders, HEMS use was associated with a mortality reduction of 33%.

**Commentary**

Propensity scoring is an increasingly-used method to allow for assessment of transport mode and trauma outcome. One of the attractions of the technique, is that overall mortality is often sufficiently low that incorporation of myriad covariates stretches the mathematical capabilities of generalized linear modeling. As the authors point out in a lucid explanation, propensity scoring allows for control of multiple confounders with a single term in the model. The model can then incorporate “standard” covariates (ISS, RTS, distance) and generate a fairly robust estimate for HEMS impact. It is noteworthy that, regardless of the fact that this study's methodology differs from most of the studies in the HEMS literature, the general estimate for scene mortality improvement falls very close to the most commonly encountered range (in the literature as a whole) of 25–30%.

## 10. Trauma-Scene and Interfacility

### 10.1. Mitchell AD, Tallon JM, Sealy B

“Air versus ground transport of major trauma patients to a tertiary trauma centre: A provincewide comparison using TRISS analysis”, *Can J Surg, *2007; 50 : 129–133.

**Objective**

The study's objective was to compare the outcomes of blunt trauma patients transported by HEMS, as compared to ground EMS, in a primarily rural, provincewide integrated trauma system with a single tertiary receiving center.

**Methods**

**Study Design**

The study was a retrospective trauma database review.

**Setting**

The setting was the Queen Elizabeth Health Sciences Centre and Dalhousie University, Halifax, Nova Scotia. The HEMS unit is staffed by a nurse/paramedic team and operates around the clock.

**Time Frame**

Study patients were those who arrived at the study hospital between 1998 and 2002.

**Patients**

Study patients were all (*n* = 791) adult (>15 years old) blunt trauma scene transports to the study center during the study period, with ISS being at least 12; 237 (30%) were transported by HEMS and 554 (70%) were transported by ground EMS. Median ISSs for air and ground patients were 25 and 20, respectively. Only 16% of HEMS transports came directly from the scene to the study center; 56% of ground EMS patients were scene transports.

**Analysis**

The analysis used TRISS. Importantly, there was a ground control group; so the performance of HEMS and ground EMS could be compared against TRISS-predicted, as well as against each other. This is important, given the opposite directionality of air and ground EMS effects on outcome (see the following).

**Results**

As compared to TRISS-predicted survival, HEMS patients had significantly better outcome—a 25% improvement in mortality as compared to predicted. Ground EMS-transported patients not only failed to have improved outcome over TRISS-predicted but also actually had significantly *higher *mortality than predicted by TRISS; in this study ground transport equated with a 10% increase in mortality. With a *W *score of 6.4, HEMS was found to result in 64 lives saved per 1000 transports. The negative *W* score of −2.4 for ground EMS indicated that there were 24 unexpected *deaths* per 1000 ground ambulance transfers. In *post hoc* analysis excluding falls, the deleterious effects of ground EMS transport disappeared: outcomes in the nonfall group for ground EMS were equal to TRISS-predicted. In the nonfall group, however, HEMS patients still had a significantly improved outcome over TRISS-predicted (*W *of 6.6 indicating 66 lives saved per 1000 transports).

**Authors' Conclusions**

This first provincewide study, focusing on a rural area, finds that HEMS transport of patients with ISS at least 12 is associated with significantly improved outcomes as compared with ground transport.

**Commentary**

As the authors themselves are quick to note, their methodology benefits from the availability of a single trauma database covering the entire province. All of the system's trauma patients are thus captured in the analysis, and the authors thus avoid the selection bias that cripples some HEMS studies [13]. In essence, the authors have conducted a population-based study, with minimization of confounding variables, in a setting ideal for detecting a HEMS benefit (a rural, maritime province with a single tertiary trauma center). If the benefit of HEMS is assessed as the difference in outcome as compared to ground transport, this study demonstrates that—in a setting admittedly ideal for HEMS benefit—air medical transport saves 88 lives per 1000 transports and decreases mortality 35%. Importantly, the study's external generalizability is improved by the fact that both scene and interfacility transports were assessed.

### 10.2. Schiller J, McCormack JE, Tarsia V, Shapiro MJ, Singer AJ, Thode HC, Henry MC

“The effect of adding a second helicopter on trauma-related mortality in a county-based trauma system”, *Prehosp Emerg Care,* 2009; 13 : 437–443.

**Objective**

The aim of this study is to compare the outcomes of adult trauma patients within a county-based trauma system, using a “natural experiment” design enabled by addition of a HEMS unit.

**Methods**

**Study Design**

The study was a before-and-after trauma database review.

**Setting**

The setting was in the state of New York. The area of interest encompasses the eastern end of Long Island, including a population of 500,000 and an area of 450 square miles. The eastern end of the service area, in the “before HEMS” period, was effectively uncovered by HEMS (due to the stationing of HEMS resources 30 miles away). In the “after” period, the county's HEMS coverage increased to include an aircraft stationed in the eastern end of Long Island. The HEMS unit is staffed by a nurse/paramedic team and operates around the clock.

**Time Frame**

Study patients were those who were injured during the 10-year period 1996–2006; the time period was split in half by the addition of the second HEMS unit in 2001.

**Patients**

Study patients were all (*n* = 1551—roughly half in each period) adult (at least 14 years old) blunt trauma patients in the county trauma registry during the study period.

**Analysis**

The primary analysis consisted of a straightforward comparison of mortality rates. Importantly, the mortality rates for all patients were assessed (including those who were not transported from the community hospitals in either period). The authors also carefully tracked, for each study period, numbers of patients who were kept in the nontrauma center hospitals and also the transport modality for those undergoing interfacility transport.

**Results**

The main study result was overall mortality, which decreased significantly in the HEMS era (dropping from 16.2% down to 11.9%, *P* = 0.02). Additionally, air transport to the regional trauma center increased by 130%, with a commensurate decrease in community (nontrauma center) hospitals' providing care for injured patients. Interestingly, interfacility HEMS transports from the community hospitals remained stable (i.e., there was no increase in HEMS utilization for interfacility transport; the increased utilization was for scene flights). The overall acuity (as measured by ISS) was not different for the HEMS period. Severely injured patients (defined by ISS at least 16) were significantly more likely to undergo HEMS transport in the HEMS period.

**Authors' Conclusions**

Introduction of a second HEMS unit to the a previously undercovered area of a county-based trauma system was associated with significantly lower trauma mortality.

**Commentary**

In a natural experiment paper covering a relatively discrete population, the authors performed an informative analysis of trauma patient care and outcomes before and after addition of dedicated HEMS to an area. The authors' methodology and discussion included many details relevant to their geography, and this paper must be read in full for an appreciation of its details. For the purposes of adding to the HEMS literature, it is a fair summary to state that the addition of HEMS resources to previously under- or uncovered areas reduces trauma mortality even in a fairly well-developed trauma system. It is more than interesting to note that the point estimate for trauma mortality reduction (26.5%) is remarkably consistent with the point estimates most often reported in other HEMS trauma literature reported on herein.

### 10.3. McVey J, Petrie D, Tallon J, Malay S, Colpitts K

“Air versus ground transport of the major trauma patient: A natural experiment”,* Prehosp Emerg Care*, 2009 (e-published ahead of print—the manuscript appeared in the print edition of *PEC, *2010; 14 : 45–50).

**Objective**

The aim of this study is to compare the outcomes of adult trauma patients transported to a Level 1 trauma center by helicopter versus ground ambulance, using a unique “natural experiment” design to obtain the ground comparison group while reducing potential confounders.

**Methods**

**Study Design**

The study was a retrospective analysis of data in two databases (the HEMS database and a provincewide trauma registry). The adult trauma patients were split into 3 groups. *Group 1* consisted of adult trauma patients transported to a tertiary care trauma center by air transport. *Group 2* patients were those triaged to HEMS (i.e., accepted by the online Medical Control Physician for air transport) but transported by ground due to aviation issues. *Group 3* included “all other” adult trauma patients transported by ground ambulance.

**Setting**

The study was a retrospective study from the largely rural Province of Nova Scotia. There is a single helicopter which serves the entire province (population about one million), executing about 600 missions per year. All provincial patients go to a single trauma center. The HEMS unit is staffed by a nurse/paramedic team and operates around the clock.

**Time Frame**

Study patients were those transferred between July 1, 1997 and June 30, 2003.

**Patients**

The study included 397 adult (at least 16 years of age) trauma patients flown by LifeFlight (*Group 1*), 57 ground-transported patients initially triaged to HEMS (*Group 2*), and 1195 patients who were initially triaged to, and subsequently transported by, ground EMS (*Group 3*).

**Analysis**

The primary outcome of interest of this study was mortality. The analyses that were performed used TRISS-based methodology.

**Results**

There was no statistically significant difference between *Group 1 *and *Group 2 *with respect to mean age, gender, percentage with blunt injury, AIS, and ISS; *Group 3 *was of lesser acuity. There was no difference in the time between injury and trauma center arrival, between *Group 1 *and *Group 2*. *Group 1 *patients had a proportion of scene calls (20%) that was higher than that of *Group 2 *(7%); *Group 3 *patients were mostly (58%) scene transports which were related in part to their being more urban in nature. As compared to *Group 2 *patients (whose mortality was equal to TRISS-predicted), *Group 1 *status was associated with statistically significant survival improvement (5.61 more lives per 100 transports). *Group 3 *patients had the worst outcome, with a survival *less* than that predicted by TRISS (*W *= −2.02).

**Authors' Conclusions**

This unique natural experiment led to methodological improvement in matching air versus ground cohorts and reduced confounding. Using naturally assigned patient groups, air transport of the adult major trauma patient in Nova Scotia is associated with significantly improved survival as compared to ground transport of similar patients.

**Commentary**

Although the study has some limitations (as reported by the authors), its message is potent given the methodological strengths of the approach and the fact that it extends (by both methodology and date) the study of Mitchell et al. from the same area [14]. First, some of the study's shortcomings should be acknowledged. The study area is largely rural, with prolonged prehospital times and only a single tertiary trauma center. The results, however internally valid they may be, are thus of uncertain external validity when considering more urban areas. The authors also identify imperfections associated with TRISS use, although they correctly point out the lack of a preferable alternative. In any even, given the particular natural experiment design used in this study, any analytic technique would have to be truly biased (e.g., give an unfair advantage to HEMS as compared to ground EMS patients) in order to lead to spurious results. There is no reason to believe that TRISS would be biased in this population.

What does this study add to the (few dozen) TRISS studies that suggest some HEMS-associated outcomes improvement? While the authors' point estimate for lives saved per 100 transports is remarkably consistent with that of the preponderance of the literature [2–4, 15], there are aspects to the current study that set it apart for methodological interest and excellence.

First, this is a provincewide study, population-based. In such a relatively small population (with only one major trauma center), this means that the selection bias that plagued some other HEMS natural experiment studies in trauma [13] and nontrauma [16] is substantially reduced. Second, the authors' concurrent natural experiment design is unique in the trauma literature and superior (in its minimization of selection bias) to the only similar study—of cardiac patients—in the HEMS nontrauma literature [16].

The use of concurrent design, in which the triage decision-making was the same for *Group 1 *and *Group 2*, is powerful. The “before-and-after” approach characterizing the other HEMS natural experiment trauma studies is useful, but potentially limited by non-HEMS trauma system changes [13, 17, 18]. The strength of the current study is enhanced by the facts that the system and patients were served by only one helicopter, with one dispatch center, and by one tertiary trauma center.

The fact that the pretrauma center times were similar in *Group 1* and *Group 2* may or may not mean that the HEMS logistics contributions were minimal in every case. However, the similarity in pretrauma center times does indicate that the time variable was not likely an overriding factor in overall survival benefit.

## 11. Trauma-Interfacility

### 11.1. Brown JB, Stassen NA, Bankey PE, Sangosanya AT, Cheng JD, Gestring ML

“Helicopters improve survival in seriously injured patients requiring interfacility transfer for definitive care”, *J Trauma,* 2011; 70 : 310–314.

**Objective**

This study's objective was to assess whether helicopter transport (HT) was associated with a survival benefit when compared with ground transport (GT) in a population of patients requiring interfacility transfer for definitive management of traumatic injury.

**Methods**

**Study Design**

This was a retrospective study using the National Trauma Databank version 8.

**Setting**

The patient sample was nationwide.

**Time Frame**

Data from 2007 were analyzed.

**Patients**

Patients transferred from a referring hospital (RH) to a trauma center by helicopter or by ground ambulance were included. Patients transferred by fixed wing aircraft were excluded.

**Analysis**

A forward stepwise logistic regression model was used to determine whether transport modality was an independent predictor of survival after adjustment for covariates. Regression analysis was repeated in subgroups with Injury Severity Score (ISS) ≤15 and ISS >15. Survival was compared in both univariate and multivariate regression analyses.

**Results**

In this study 74,779 subjects were transported by helicopter (20%) or ground (80%). Mean ISS was higher in HT patients (17 ± 11 versus 12 ± 9, *P* < 0.01), as was the percentage of patients with ISS > 15 (49% versus 28%; OR 2.53, 95% CI 2.43 to 2.63). HT had higher rates of intensive care unit admissions (54% versus 29%; OR 2.86, 95% CI 2.75 to 2.96), mechanical ventilation (25% versus 9%; OR 3.49, 95% CI 3.33 to 3.66), and requirement for emergent surgical intervention (19% versus 13%; OR 1.52, 95% CI 1.43 to 1.60). Univariate analysis showed that overall survival was lower in HT patients as compared to GT patients (92% versus 96%, *P* < 0.01). In subgroup analysis overall survival for transfer patients with ISS ≤15 was also slightly lower in the HT group (98% versus 99%; *P* = 0.01). Overall survival for transfer patients with ISS >15 was again lower in HT group (85% versus 90%; *P* < 0.01). However, HT became an independent predictor of survival to discharge after adjusting for covariates (OR 1.09, 95% CI 1.02 to 1.17, *P* = 0.01) in patients with ISS >15.

**Authors' Conclusions**

In this first national-level study to compare HT and GT with respect to interfacility trauma patients' outcomes, HT appears to convey a survival benefit in those patients who are more severely injured. HT was an independent predictor of survival in patients with ISS >15. However, HT was not an independent predictor of survival in less severely injured patients (ISS ≤15). HT offered shorter transport and overall prehospital times. Those patients being transferred by helicopter had consistently higher injury severity markers. HT patients utilize a higher level of hospital resources and the data support a contention that, at a national level, providers are triaging more severely injured patients for HT.

**Commentary**

The largest ground-versus-air transport comparison in the interfacility trauma literature (HEMS *n* 14,771, GEMS *n* 60,008), this analysis of National Trauma Data Bank (NTDB) data followed the same general lines as a scene trauma paper by the same authors (published a few months earlier, in December 2010's *J Trauma*); the difference was that this paper assessed interfacility transports. The authors' overall multivariate analysis incorporated the myriad covariates described above for the scene run paper. Multivariate regression reported was reported as negative for demonstrating HEMS survival benefit; the point estimate of 6% improvement in OR (point estimate 1.06) was associated with a 95% CI that just crossed the null value (0.99 to 1.13, *P* = 0.07). The authors also executed an *a priori *planned subgroup analysis on those patients with ISS below and those with ISS above, a cutoff of 15. For those patients with lower ISS, HEMS was (unsurprisingly) associated with no survival benefit (OR point estimate and 95% CI: 1.06, 0.92 to 1.24, *P* = 0.42). For patients with more serious injury—which group constituted 49% of all HEMS transports—there was a significant mortality improvement associated with air medical transport (OR 1.09, 95% CI 1.02 to 1.17, *P* = 0.01). HEMS patients were far more severely injured (e.g., intra- and early posttransport deaths 10x higher) and required substantially more resources (e.g., 50% more likely to need emergency operation), than those transported by ground EMS. The study's broad array of covariates and the clear demonstration of improved outcome in a group (those with ISS exceeding 15) comprising half of the air-transported cohort adds to the strength of the study message. The study's authors pointed out additional interesting facts addressing logistics and utilization (e.g., only 8% of HEMS patients were discharged within 24 hours). Among the study weaknesses acknowledged by the authors was the failure to account for possible morbidity improvements that could benefit patient with lesser injury acuity.
